# Microbial dysbiosis and the host airway epithelial response: insights into HIV-associated COPD using multi’omics profiling

**DOI:** 10.1186/s12931-023-02431-4

**Published:** 2023-05-04

**Authors:** Marcia Smiti Jude, Chen Xi Yang, Fernando Studart Leitao Filho, Ana I. Hernandez Cordero, Julia Yang, Tawimas Shaipanich, Xuan Li, David Lin, Julie MacIsaac, Michael S. Kobor, Sunita Sinha, Corey Nislow, Amrit Singh, Wan Lam, Stephen Lam, Silvia Guillemi, Marianne Harris, Julio Montaner, Raymond T. Ng, Christopher Carlsten, S. F. Paul Man, Don D. Sin, Janice M. Leung

**Affiliations:** 1 Centre for Heart Lung Innovation, St. Paul’s Hospital, Centre for Heart Lung Innovation, University of British Columbia, Room 166-1081 Burrard St., Vancouver, BC V6Z 1Y6 Canada; 2 Division of Respiratory Medicine, Department of Medicine, University of British Columbia, Vancouver, BC Canada; 3 Centre for Molecular Medicine and Therapeutics, University of British Columbia, Vancouver, BC Canada; 4 Faculty of Pharmaceutical Sciences, University of British Columbia, Vancouver, BC Canada; 5 British Columbia Cancer Research Centre, Vancouver, BC Canada; 6 British Columbia Centre for Excellence in HIV/AIDS, Providence Health Care, Vancouver, BC Canada; 7 Faculty of Medicine, University of British Columbia, Vancouver, BC Canada; 8 Department of Computer Science, University of British Columbia, Vancouver, BC Canada

**Keywords:** HIV, COPD, Microbiome, Airway epithelium, Methylation, Transcriptome, Epigenetic

## Abstract

**Background:**

People living with HIV (PLWH) are at increased risk of developing Chronic Obstructive Pulmonary Disease (COPD) independent of cigarette smoking. We hypothesized that dysbiosis in PLWH is associated with epigenetic and transcriptomic disruptions in the airway epithelium.

**Methods:**

Airway epithelial brushings were collected from 18 COPD + HIV + , 16 COPD − HIV + , 22 COPD + HIV − and 20 COPD – HIV − subjects. The microbiome, methylome, and transcriptome were profiled using 16S sequencing, Illumina Infinium Methylation EPIC chip, and RNA sequencing, respectively. Multi ‘omic integration was performed using Data Integration Analysis for Biomarker discovery using Latent cOmponents. A correlation > 0.7 was used to identify key interactions between the ’omes.

**Results:**

The COPD + HIV −, COPD −HIV + , and COPD + HIV + groups had reduced Shannon Diversity (p = 0.004, p = 0.023, and p = 5.5e−06, respectively) compared to individuals with neither COPD nor HIV, with the COPD + HIV + group demonstrating the most reduced diversity. Microbial communities were significantly different between the four groups (p = 0.001)*.* Multi ‘omic integration identified correlations between *Bacteroidetes Prevotella*, genes *FUZ, FASTKD3*, and *ACVR1B*, and epigenetic features *CpG-FUZ* and* CpG-PHLDB3.*

**Conclusion:**

PLWH with COPD manifest decreased diversity and altered microbial communities in their airway epithelial microbiome. The reduction in *Prevotella* in this group was linked with epigenetic and transcriptomic disruptions in host genes including *FUZ, FASTKD3,* and *ACVR1B.*

**Supplementary Information:**

The online version contains supplementary material available at 10.1186/s12931-023-02431-4.

## Background

Antiretroviral therapy (ART) has substantially increased the lifespan and reduced acquired immunodeficiency syndrome-related morbidity and mortality of people living with human immunodeficiency virus (PLWH, HIV) [1, 2]. The aging of PLWH nonetheless places them at higher risk for developing age-related comorbidities, including chronic obstructive pulmonary disease (COPD). PLWH have been shown to have an elevated risk for COPD [3–5] which appears to be independent of smoking history [3, 6] and also worsens their mortality risk [7]. As the population of PLWH ages, they are likely to bear an increasing burden of COPD.

There are many hypotheses regarding the mechanisms of HIV-associated COPD, including but not limited to longer exposure to risky behaviours [8], side effects from ART [8, 9], chronic inflammation [10], and *Pneumocystis* colonization [11, 12]. Speculation that microbial dysbiosis in the lung, the result of repeated pulmonary infections and antibiotic exposure, could also drive obstructive lung disease has led to multiple studies investigating the lung microbiome in PLWH [13–16]. For instance, we previously reported on decreased microbial diversity and community shifts in the airway epithelium of PLWH compared to HIV-uninfected patients [16]. The characterization of the airway epithelial microbiome specifically in PLWH with COPD, however, has yet to be reported. Moreover, although dysbiosis may conceivably lead to profound changes in host molecular mechanisms such as transcription, epigenetic regulation, metabolism, and immunity, these disruptions have not yet been fully described.

In this study, we hypothesize that microbial disruptions in the airway epithelium of PLWH with COPD are associated with key transcriptomic and epigenetic alterations that can help us gain insights into the disease pathogenesis of HIV-associated COPD. We simultaneously profile the microbiome, methylome, and transcriptome from airway epithelial cells collected via bronchoscopy in PLWH with COPD (COPD + HIV +) to (1) characterize the distinct microbiome features distinguishing them from PLWH without COPD (COPD − HIV +), HIV-uninfected patients with COPD (COPD + HIV−), and healthy controls (COPD − HIV−) and (2) link these features with epigenetic and transcriptomic alterations to better understand the host response to dysbiosis. To the best of our knowledge, no study has integrated and examined all three ’omes together in the context of the HIV airway.

## Methods

### Study population and design

Airway epithelial cell (AEC) brushings were obtained from 76 (18 COPD + HIV +, 16 COPD− HIV + , 22 COPD + HIV− and 20 COPD – HIV −) adult patients at St. Paul’s Hospital, Vancouver, BC, who consented to undergo bronchoscopic collection of research specimens under the University of British Columbia Research Ethics Board Certificates H11-02713 and H15-02166. Bronchial brushings were obtained according to previously published methods [16–18]. Briefly, the bronchoscope was guided to either the right or left upper lobe segment and a cytology brush was inserted until resistance was met (approximately 2 mm in diameter) at which point gentle brushings were obtained for AEC collection. PLWH also provided background controls for the study, including oral washings, reagent controls, bronchoscope channel washings, water rinsed over unused cytology brushes, and extraction negative samples specifically to investigate for any cross-contamination with AEC samples. COPD was defined based on a pulmonologist’s diagnosis of COPD and either a pre-bronchodilator forced expiratory volume in one second (FEV_1_)/forced vital capacity (FVC) ≤ lower limit of normal [19] or clear evidence of emphysema on computed tomography imaging on visual inspection. PLWH were defined as subjects with documented HIV-1 infection. An overview of the study design is provided in Additional file 1: Fig. S1. Details of the cohort and the methylation and transcriptome profiling of the samples have previously been reported [18]. Briefly, methylation profiles were obtained using the Illumina Infinium MethylationEPIC BeadChip microarray, which captures the methylation status 863,904 CpG sites across the genome. Paired end RNA sequencing was performed to a depth of 50 million reads using the Illumina Novaseq6000 platform.

### Microbiome profiling

Bacterial genomic DNA was extracted from bronchial brushings, and microbiome profiles were obtained using touchdown droplet digital polymerase chain reaction, followed by 16S amplicon sequencing using the Illumina Miseq^®^ platform at the Sequencing and Bioinformatics Consortium at the University of British Columbia (Vancouver, Canada). Sequencing data were processed following the QIIME2™ workflow using Divisive Amplicon Denoising Algorithm (DADA2) to denoise sequences. During these steps, the sequencing reads were demultiplexed, merged and resolved into amplicon sequence variants (ASVs). Further quality filtering steps were performed to remove contaminating host mitochondrial or chloroplast sequences, ASVs present in PCR controls, ASVs with significantly fewer sequences than the majority, and ASVs present only in one sample (singletons). Taxonomy assignment was performed on ASVs using a pre-trained naive Bayes classifier artifact trained against Greengenes (138 revision) trimmed to include only the V4 hypervariable region and pre-clustered at 99% sequence identity. Phylogenetic trees were generated using the MAFFT program in QIIME 2™, which was consecutively used as input to compute different phylogenetic diversity measures.

Alpha diversity was measured using the Shannon Diversity Index, a metric of community richness and evenness. T-tests were used to identify differences in Shannon Diversity between PLWH and HIV-uninfected groups and between COPD and non-COPD groups, while analysis of variance (ANOVA) and Mann–Whitney U-tests were used to identify differences between the COPD-HIV-, COPD + HIV −, COPD −HIV + , and COPD + HIV + groups. Beta diversity was measured using Bray Curtis Dissimilarity, which measures differences in richness between two or more communities, tested with permutational multivariate analysis of variance (PERMANOVA) (adonis function in vegan R package [20]), and visualized using principal coordinate analysis (PCoA) plots. For both alpha and beta diversity, a second model was performed to adjust for age, sex, and smoking status using the following: Diversity Metric ~ Age + Sex + Smoking Status + COPD/HIV. We also examined the interaction effects between COPD and HIV on alpha and beta diversity metrics. Average relative taxon abundance comparisons were performed between the COPD + HIV + , COPD −HIV + , COPD + HIV− and COPD −HIV − groups at the phylum and genus levels. The Kruskal–Wallis and Dunn’s tests were used to identify between group differences. Significant taxon differences were identified at false discover rate (FDR) < 0.05 using the Benjamini–Hochberg method.

### Multi ’omic integration

The microbiome, transcriptome, and methylome were integrated using Data Integration Analysis for Biomarker discovery using Latent cOmponents (DIABLO) implemented in the mixOmics R package. This method simultaneously identifies key ’omics features among heterogeneous datasets and their respective correlations [21, 22].

This integration analysis included features from the three datasets as input: microbiome (126 ASVs), transcriptome (28 genes) and methylome (4404 CpGs). The ASVs were obtained after quality filtering and taxonomy assignment steps described above. Top genes and CpG sites were selected based on robust linear modeling examining the interaction effect of COPD*HIV on gene expression and methylation, respectively (further methods provided in the Additional file 1: Methods). The following design matrix with values ranging between 0 (indicating no correlation between ’omics datasets) to 1 (indicating maximum correlation) was chosen, such that there was a compromise between correlation and discrimination between the features across the different datasets.

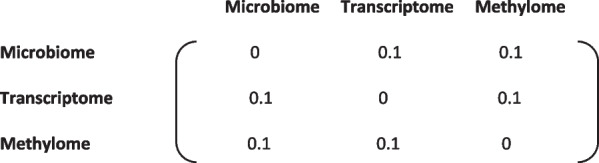



Subsequently, a DIABLO model with no variable selection was fit to evaluate the global performance and choose the number of components (ncomp) for the final model. The ncomp were chosen with considerations of centroids distance measures and the balanced error rates, after tenfold cross validation repeated 50 times. Sparse partial least squares discriminant analysis was then used to obtain the optimal number of variables of each component in the three datasets, after tenfold cross validation repeated 50 times. Using the chosen parameters, the final DIABLO models were fit to identify key interactions (using a correlation threshold of |0.7|) between the microbiome, methylome and the transcriptome. Similar methods were used to integrate just two datasets at a time—the (a) microbiome and methylome, and (b) microbiome and transcriptome—to identify correlations between the corresponding ’omes.

## Results

Table 1 provides a summary of participant demographics. The study cohort was composed of 76 participants (18 COPD + HIV + , 16 COPD − HIV + , 22 COPD + HIV −, and 20 COPD – HIV −). The mean (standard deviation) age was 61.3 ± 11.6 years, with males (n = 48) making up 63.2% of the total. Of the PLWH (n = 34), 30 (88.2%) were receiving ART and 26 (76.5%) had undetectable HIV plasma viral load (< 40 copies/mL), and the mean CD4 count was 439 cells/mm^3^.

**Table 1:** Demographics and clinical features of the study cohort

	HIV + COPD +	HIV + COPD −	HIV − COPD +	HIV − COPD −	P*
n	18	16	22	20	*–*
Age (years)	56 (52–63)	56 (53–61)	69 (64–73)	64 (56–68)	*1.02* × *10*^*–03*^
Females (%)	22	13	45	60	*0.01*
BMI (kg/m^2^)	24.06 (19.16–26.68)	26.81 (23.09–27.52)	24 (22.86–29.50)	25.25 (21.4–29.39)	*0.17*
Smoking status	–	–	–	–	*1.58* × *10*^*–04*^
Current (%)	61	19	40	5	–
Former (%)	28	50	55	35	–
Never (%)	5.5	25	5	60	–
Unreported (%)	5.5	6	0	0	
Pack-year history	38 (30–49)	4.50 (0.75–23.25)	40 (20–50)	20 (0–30)	*2.02* × *10*^*–04*^
Bronchiectasis (%)	11	25	0	5	–
Asthma (%)	0	25	5	0	–
Pre FEV_1_%	80.80 (55.55–90.10)	83.00 (78.70–91.50)	70.00 (59.88–78.53)	88.75 (81.50–96.25)	*3.47* × *10*^*–03*^
Pre FEV_1_/FVC	64.61 (59.06–75.36)	75.00 (71.64–80.59)	63.45 (59.93–69.50)	75.90 (72.85–81.25)	*3.46* × *10*^*–06*^
Undetectable HIV viral load (%)	89	63	–	–	*0.16*
CD4 T cell count (cells/mm^3^)	450.0 (260.0–510.0)	460.0 (110.0–620.0)	–	–	*0.91*
On ART (%)	94	81	–	–	*0.32*

Quantitative variables are described with the median and interquartile range “()”

*Differences between the groups’ characteristics were tested using Kruskal–Wallis test for continuous variables and a X-squared test for count variables. “Pre” refers to spirometry tests before bronchodilator use. Denominators used for the percentages correspond to the total number of individuals in each group

### PLWH with COPD feature reduced airway epithelial microbiome diversity and microbial community shifts

**Fig. 1:**
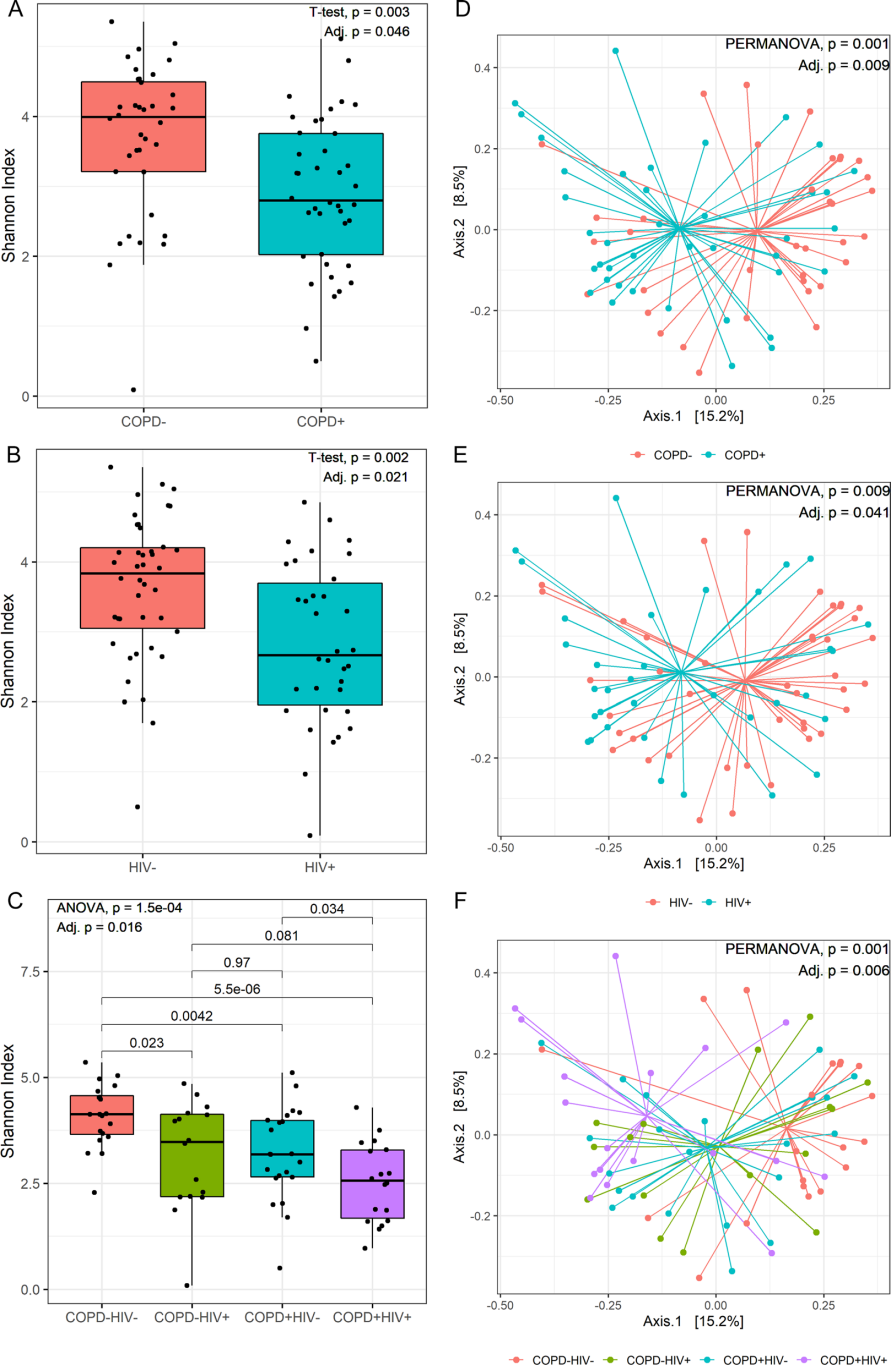
Shannon Diversity Index differences between (**a**) COPD + and COPD− groups, (**b**) HIV + and HIV− groups, and (**c**) COPD + HIV + , COPD + HIV−, COPD−HIV + and COPD−HIV− groups. The COPD + HIV + group featured the lowest Shannon Diversity Index of all four groups. P-values were adjusted for age, sex, and smoking status. Microbial community structures in AECs according to (**d**) COPD status (COPD− (N): red circles; COPD + (Y): blue circles), (**e** HIV status (HIV− (Negative): red circles; HIV + (Positive): blue circles), and (**f**) combined COPD + HIV status (COPD + HIV +: purple circles; COPD + HIV−: blue circles; COPD-HIV +: green circles; COPD−HIV−: red circles) based on Bray–Curtis distances; the centroids for each group are also shown. Permutational multivariate ANOVA (PERMANOVA) was used for comparisons of microbial community structures between groups, with adjustment for age, sex, and smoking status

There were no significant differences in 16S rRNA gene copy levels between the COPD + HIV + , COPD + HIV −, COPD − -HIV + and COPD – HIV − groups (overall Kruskal–Wallis p = 0.300, Additional file 1: Fig. S2). Alpha diversity measured using the Shannon Diversity Index is represented in Fig. 1A–C. We observed significant differences between the (a) COPD + and COPD − groups (median[interquartile range] 2.80[1.73] vs. 3.99[1.29]; p = 0.003, adjusted p = 0.046), (b) HIV + and HIV − groups (2.66[1.74] vs. 3.83[1.15]; p = 0.002, adjusted p = 0.021), and (c) combined COPD and HIV groups: COPD + HIV + , COPD + HIV −, COPD − HIV + and COPD – HIV − (2.56[1.61] vs. 3.19[1.33] vs. 3.48[1.94] vs. 4.13[0.91]; p = 1.5e−04, adjusted p = 0.016). PLWH with COPD featured the lowest alpha diversity of the four groups. There were no statistically significant differences in alpha or beta diversity measures by sex groups.

Beta diversity measured between the COPD −HIV −, COPD + HIV −, COPD −HIV + , and COPD + HIV + groups are shown in Fig. 1D–F. The principal coordinate plots and PERMANOVA analysis show that there were significant differences between the resident microbial communities of the COPD + and COPD − groups (p = 0.001, adjusted p = 0.009), HIV + and HIV − groups (p = 0.009, adjusted p = 0.041), and the combined COPD and HIV groups (p = 0.001, adjusted p = 0.006). Consistent with these findings, smoking status was also associated with significant differences in resident microbial communities (p = 0.037, Additional file 1: Fig. S3). There were no significant interaction effects between COPD and HIV on alpha and beta diversity metrics (Additional file 1: Fig. S4). Removal of patients using inhaled corticosteroids (n = 4) did not significantly change relationships in alpha and beta diversity (Additional file 1: Figs. S5, S6).

**Fig. 2:**
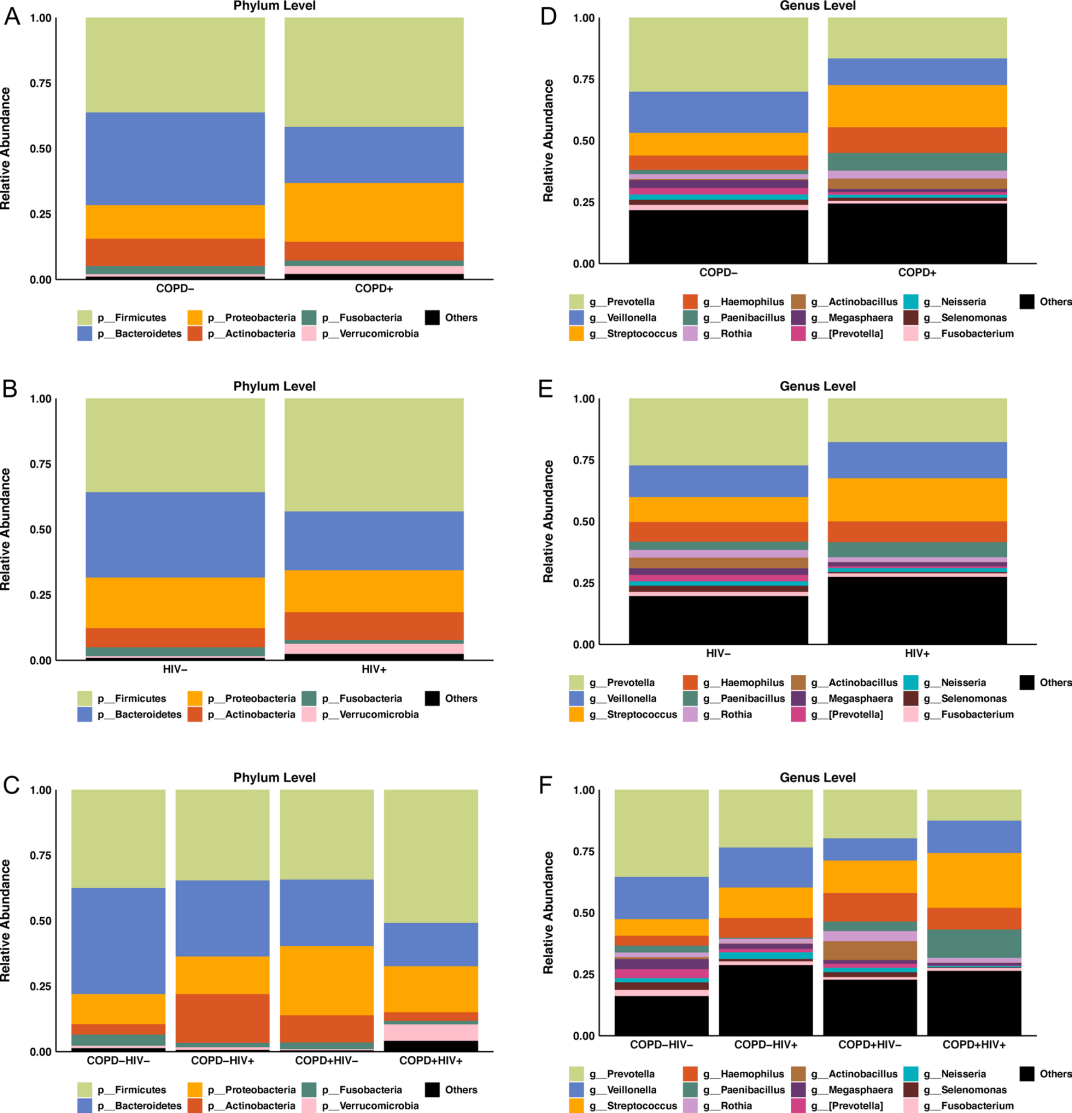
Average relative taxa abundance comparisons at the phylum level between (**a**) COPD + and COPD− groups, (**b**) HIV+ and HIV− groups, and (**c**) COPD + HIV + , COPD + HIV−, COPD−HIV + and COPD−HIV− groups. Average relative taxa abundance comparisons at the genus level between (**d**) COPD + and COPD− groups, (**e**) HIV + and HIV− groups, and (**f**) COPD + HIV + , COPD + HIV−, COPD−HIV + and COPD−HIV− groups

Relative phyla and genera abundance are shown in Fig. 2 and in Additional file 1: Tables S1 and S2. The most abundant phylum in airway epithelial cells was *Firmicutes,* followed by *Bacteroidetes* and *Proteobacteria*. At the genus level, *Prevotella, Veillonella*, and *Streptococcus* were the most abundant. COPD − showed higher relative abundance of phyla *Bacteroidetes* and *Fusobacteria, and* genera *Prevotella, Veillonella, Megasphaera, Neisseria, Selenomonas*, and *Fusobacterium* compared to the COPD + group*.* Similarly, the HIV − group had higher abundance of phyla *Fusobacteria,* and genera *Prevotella, Neisseria, Selenomonas and Fusobacterium* compared to the HIV + group. Phyla *Fusobacteria* and *Bacteroidetes*, and genera *Prevotella, Megasphaera, Neisseria, Selenomonas* and *Fusobacterium* showed significant differences between the 4 groups (COPD-HIV-, COPD-HIV +, COPD + HIV −, and COPD + HIV +). There were no individual genera or phyla, however, that were significantly correlated with FEV_1_ percent predicted.

To address possible microbial contamination in AEC brushings, oral washings, reagent controls, bronchoscope channel washings, water rinsed over unused cytology brushes, and extraction negative samples were collected from the PLWH group. 16S rRNA gene copies were significantly elevated in the AEC brushings and oral wash controls compared to the background environmental samples (Additional file 1: Fig. S7A). PCA plots demonstrated that each sample type had a distinct community profile (Additional file 1: Fig. S7B, Table S3), thus suggesting that there was minimal cross-contamination.

### Multi ‘omic integration using DIABLO

**Fig. 3:**
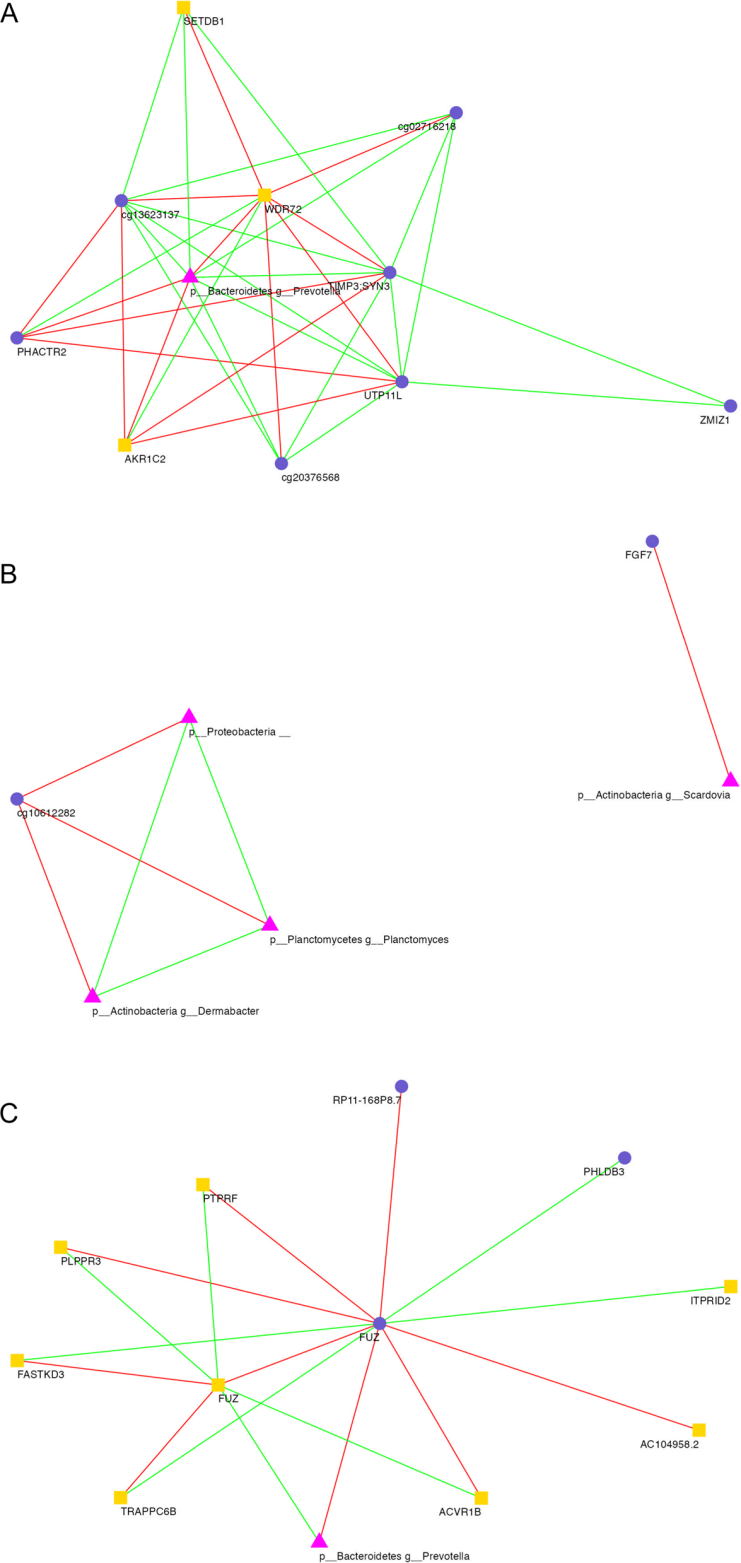
Network visualization of correlations between the microbiome, methylome and transcriptome in (**a**) COPD, (**b**) HIV, and (**c**) COPD*HIV analyses. Nodes represent multiomic features (ASVs—Pink triangle, CpGs—Purple circle, and genes—Yellow square), and edges connecting any two nodes corresponds to their correlation (Positive correlation—Green; Negative correlation—Red). Correlation cutoff = 0.75

The microbiome, transcriptome and methylome were integrated using DIABLO to identify any ‘between’ and ‘within’ ’ome correlations based on (a) COPD, (b) HIV and (c) COPD*HIV statuses (Additional file 1: Fig. S8). The top three ASV-Gene, ASV-CpG and Gene-CpG pairs and their respective correlation values are shown in representative Table 2. The balanced error rate, used as an estimate of model performance, was ∼30–40% in each component. Network interactions between the microbiome, methylome, and transcriptome for the strongest correlations (> 0.75) are shown in Fig. 3. Complete tables of pairwise correlations for COPD, HIV, and COPD*HIV are provided in Additional files 2, 3, and 4, respectively.

**Table 2:** Top ASV-Gene, ASV-CpG and Gene-CpG pairs and their respective correlation values

	Correlation
(a) COPD effect	
Top ASV-gene pairs	
* p Bacteroidetes g Prevotella* – *WDR72*	− 0.797
* p Bacteroidetes g Prevotella* – *AKR1C2*	− 0.765
* p Bacteroidetes g Prevote*lla – *SETDB1*	0.761
Top ASV-CpG pairs	
* p Bacteroidetes g Prevotella*—CpG *TIMP3;SYN3*	0.7993
* p Bacteroidetes g Prevotella*—CpG *UTP11L*	0.791
* p Bacteroidetes g Prevotella*—CpG *PHACTR2*	− 0.760
Top Gene-CpG pairs	
* WDR72*—CpG *TIMP3;SYN3*	− 0.799
* WDR72*—CpG *UTP11L*	− 0.784
* AKR1C2*—CpG *UTP11L*	− 0.775
(b) HIV effect	
Top ASV-CpG pairs	
* p Actinobacteria g Scardovia—*CpG* FGF7*	− 0.811
* p Proteobacteria—*CpG* ABCF3*	− 0.706
* p Planctomycetes g Planctomyces—*CpG *ABCF3*	− 0.706
(c) COPD*HIV Effect	
Top ASV-gene pairs	
* p Bacteroidetes g Prevotella*—*FUZ*	0.774
* p Bacteroidetes g Prevotella—FASTKD3*	− 0.747
* p Bacteroidetes g Prevote*lla—*ACVR1B*	0.747
Top ASV-CpG pairs	
* p Bacteroidetes g Prevotella*—CpG *FUZ*	− 0.785
* p Bacteroidetes g Prevotella*—CpG *RP11-168P8.7*	0.715
* p Bacteroidetes g Prevotella*—CpG *PHLDB3*	− 0.707
Top Gene-CpG pairs	
* WDR72—*CpG* FUZ*	− 0.819
* WDR72—*CpG* FUZ*	− 0.800
* AKR1C2—*CpG* FUZ*	− 0.793

(a) COPD effect, (b) HIV effect, and (c) COPD*HIV effect. Correlation cutoff = 0.7

In both our COPD effect and combined COPD*HIV effect analyses, we identified *Bacteroidetes Prevotella* to be a top ASV correlated with features of the transcriptome and the epigenome. In the former analysis, *Bacteroidetes Prevotella* was correlated with genes *WDR72, AKR1C2* and *SETDB1,* and methylation sites CpG-*TIMP3;SYN3,* CpG-*UTP11L* and CpG-*PHACTR2;* CpG-*UTP11L* was in turn correlated with genes *WDR72* and *AKR1C2*, and CpG-*TIMP3; SYN3* was correlated with gene *WDR72.* In the COPD*HIV analysis, *Bacteroidetes Prevotella* was correlated with genes *FASTKD3, FUZ,* and *ACVR1B*, and CpG-*FUZ* and CpG-*PHLDB3*.

## Discussion

In support of the hypothesis that HIV infection is associated with alterations in the airway epithelial microbiome, our analysis showed several novel conclusions: (1) PLWH with COPD had significantly lower microbial diversity and a distinct microbial community in their airway epithelium compared to PLWH without COPD, HIV-negative COPD patients, and patients with neither HIV nor COPD; (2) microbial features that appeared disrupted in PLWH with COPD were correlated with methylation and transcriptomic alterations along genes not previously recognized to be part of the pathogenesis of HIV-associated COPD.

Our analysis of the microbiome revealed that relatively “healthier” individuals were enriched in characteristic phyla *Fusobacteria* (in COPD −, HIV − and COPD – HIV − groups) and *Bacteroidetes* (in the COPD − and COPD −HIV − groups), and that decreases in these phyla may be associated with disease. However, we found no significant differences in the relative abundance of other characteristic phyla *Proteobacteria* and *Firmicutes*, contrary to the findings of Sze et al. [23], Xu et al.[16], and Ramsheh et al. [24]. Lower in the taxonomic hierarchy, we noted differences in relative abundance of genera *Prevotella, Selenomonas, Neisseria, Fusobacterium, Streptococcus and Veillonella* in the group with both COPD and HIV*.* These microbes are known to be oral commensals and have previously been implicated in lower airway colonization and driving severity of disease in COPD [25–27]. Our results reinforce the notion that diversity and composition are important components of a “healthy” microbiome [28]. However, how exactly lung microbial dysbiosis translates to the clinical disease presentations observed in patients with HIV and COPD is still unclear. This relationship is likely multifactorial, with variations in host processes such as transcription, metabolism, and immunity contributing to a certain extent.

In light of this uncertainty, we carried out multi ‘omic integration to effectively combine information from three ‘omes (microbiome, methylome and transcriptome) and identified highly correlated ‘omic features that may be relevant in the combined COPD and HIV states. Our integrative analyses consistently identified the single microbiome feature *Bacteroidetes Prevotella*, reduced in relative abundance in PLWH with COPD. Although a widely studied microbe, the exact role of *Prevotella* in the respiratory system is still poorly understood. Certain pathobiontic strains have been implicated as promoters of subclinical inflammation, particularly through increased Th-17 cytokine expression [29]. Twigg et al.observed that long-term ART use (> 3 years), which would normally be associated with a “healthier” phenotype, was associated with decreased *Prevotella* abundance in nine PLWH [14]. On the other hand, *Prevotella* has also been described in terms of healthy microbial ecosystems. Within HIV-uninfected patients with COPD, airway epithelial abundance of *Prevotella* has been associated with increased lung function, reduced dyspnea scores and inflammation, and expression of epithelial genes involved in tight junction promotion [24]. Many of these properties of *Prevotella* may be due to its interactions with other microbes and its dynamic role within the respiratory ecosystem. Recent studies have shown that *Prevotella* may exert its anti-inflammatory effects by inhibiting cytokine production by other gram-negative bacteria like *Haemophilus influenzae* or may co-aggregate and form heterotrophic biofilms with microbes like *Porphyromonas* [30, 31]*.* Future research based on cell culture models can help determine the strain-specific phenotypic response of the host.

The power of multi ‘omics integration allows for greater insight into what impact microbial dysbiosis might have on the host airway response. For instance, *Bacteroidetes Prevotella* was highly correlated with the methylation and expression of specific genes in the COPD and COPD*HIV interaction effect multi ‘omic analyses. Moreover, we found that in PLWH with COPD, reduced *Prevotella* abundance was correlated with the increased methylation of CpG sites along the genes *PHLDB3* and *FUZ,* and the decreased expression of gene *FUZ* and the increased expression of gene *FASTKD3*. The consistent appearance of *FUZ* in relation to *Prevotella* abundance in our multi ‘omic integration analyses suggests a strong relationship between these two features along both epigenetic and transcription pathways. *FUZ* encodes a planar cell polarity protein that plays a prominent role in ciliogenesis [32]. Within the lung, higher methylation and lower expression of *FUZ* has previously been associated with tumour promotion and poor prognosis in lung adenocarcinoma [33], a compelling finding given the known increased rate of lung cancer in PLWH [34]. In our analysis, *FUZ* expression was also positively correlated *ACVR1B* and *PTPRF* and inversely correlated with *FASTKD3. ACVR1B*, part of the transforming growth factor-beta family, has established associations with COPD pathogenesis, as an identified expression quantitative trait loci in COPD blood, sputum, and lung [35, 36] and as a causal gene in emphysema distribution [37]. While the associations of *PTPRF* and *FASTKD3* with COPD are not as clear, links between these two genes and lung cancer prognosis [38, 39] suggest some degree of activity within the lung through their roles in apoptosis, cell growth and differentiation, and oncogenesis. Interestingly, *PTPRF* has been noted to have a role in regulating the assembly and contraction of actin and actomyosin, and formation of tight junctions, with potential barrier function against HIV entry into target cells [40]. Whether modulation of *Prevotella* abundance within the airway epithelium can subsequently affect the activity of these downstream genes in PLWH with COPD is unknown, but may be a worthy area for future study.

Although this study is one of the largest to evaluate the airway epithelial microbiome in HIV-associated COPD, it has several limitations. We showed that microbial disruptions are evident in the airways of PLWH with COPD, however this study was not designed to prove causation of airway injury. Future experimentation using cell culture models or germ-free animals mimicking HIV infection may provide greater insight into the direction of the microbe-gene relationships identified in this study. Second, since the majority of our cohort were receiving ART and had undetectable HIV plasma viral loads, these results may not reflect microbiome and gene changes observed in PLWH not on ART. Third, we collected brushings from only one upper lobe lung segment per patient and acknowledge that there may be regional variation in the microbiome which we would not have been able to detect. Fourth, we did not have independently verified records of the cohort’s previous exacerbation and antibiotic use history. Fifth, there were demographic differences between our study groups and future studies with greater numbers of female PLWH would be welcomed. Greater balance of concurrent asthma and bronchiectasis, as well as of lung function amongst the COPD subgroups, would also be beneficial. Finally, while a promising field to uncover novel biologic relationships, multi ‘omic integration brings further challenges. Different ‘omics datasets are often generated via varied technologies and platforms and the search for a “gold-standard” workflow for data filtering, normalization, and integration continues [21]. “Over-fitting” the data in these workflows is often a concern which can cause the predictive performance to suffer in other cohorts. Future work using a validation dataset can improve these shortcomings and provide better assurance of model performance [41].

## Conclusions

In this study, we are able to demonstrate that multi ‘omics integration can yield new insights into the impact microbial dysbiosis can have in the airway epithelium of PLWH, identifying novel genes in the pathogenesis of HIV-associated COPD. These genes could be further explored as potential biomarkers or drug targets specific to PLWH.

## Supplementary Information

**Additional file 1. Figure S1:** Multi ‘omic integration study design. **Figure S2:** 16S RNA gene copies/μL measured in airway epithelial cells. **Figure S3:** Principal coordinate plot by smoking status. **Figure S4:** Plot showing potential interactive effects of COPD and HIV status on Shannon Index, Faith PD, Bray Curtis PC1, and Bray Curtis PC2. **Figure S5:** Shannon Diversity Index differences between the COPD and HIV groups after removing 4 COPD- subjects administered with inhaled corticosteroids from the analysis. **Figure S6:** Principal coordinate plot showing microbial community structures among subject groups based on the Bray-Curtis metric, after removing 4 COPD- subjects administered with inhaled corticosteroids from the analysis. **Figure S7:** 16S RNA gene copies/μL measured in bronchial brushings and control specimens of HIV+ subjects. **Table S1** Relative taxa abundance comparisons at the phylum level between the COPD+ and COPD-,HIV+ and HIV-, andCOPD-HIV-, COPD-HIV+, COPD+HIV- and COPD+HIV+ groups in AEC samples. **Table S2:** Relative taxa abundance comparisons at the genus level between the COPD+ and COPD-, HIV+ and HIV-, and COPD-HIV-, COPD-HIV+, COPD+HIV- and COPD+HIV+ groups in AEC samples. **Table S3:** Pairwise PERMANOVA comparisons between the different specimen types obtained from HIV+ subjects.

**Additional file 2.** Containing DIABLO results by COPD status.

**Additional file 3.** Containing DIABLO results by HIV status.

**Additional file 4.** Containing DIABLO results by COPD and HIV status.

## Data Availability

Microbiome sequencing data have been deposited into the National Center for Biotechnology Information’s Sequence Read Archive (SRA) (BioProject PRJNA965797). Methylation and transcriptome data have been deposited into the GEO database (GSE178809).

## Abbreviations

AEC: Airway epithelial cell
ART: Antiretroviral therapy
ANOVA: Analysis of variance
ASV: Amplicon sequence variants
COPD: Chronic obstructive pulmonary disease
DIABLO: Data Integration Analysis for Biomarker discovery using Latent cOmponents
FDR: False discovery rate
FEV_1_: Forced expiratory volume in one second
FVC: Forced vital capacity
HIV: Human immunodeficiency virus
PCoA: Principal coordinate analysis
PERMANOVA: Permutational multivariate analysis of variance
PLWH: People living with HIV
