# Supplementary material for: Microbial dysbiosis and the host airway epithelial response: insights into HIV-associated COPD using multi’omics profiling
Source: Respir Res. 2023 May 4;24:124. doi: 10.1186/s12931-023-02431-4 (PMC10161506; doi:10.1186/s12931-023-02431-4)
Supplement: Supplementary file 1 — Additional file 1. Figure S1: Multi ‘omic integration study design. Figure S2: 16S RNA gene copies/μL measured in airway epithelial cells. Figure S3: Principal coordinate plot by smoking status. Figure S4: Plot showing potential interactive effects of COPD and HIV status on Shannon Index, Faith PD, Bray Curtis PC1, and Bray Curtis PC2. Figure S5: Shannon Diversity Index differences between the COPD and HIV groups after removing 4 COPD- subjects administered with inhaled corticosteroids from the analysis. Figure S6: Principal coordinate plot showing microbial community structures among subject groups based on the Bray-Curtis metric, after removing 4 COPD- subjects administered with inhaled corticosteroids from the analysis. Figure S7: 16S RNA gene copies/μL measured in bronchial brushings and control specimens of HIV+ subjects. Table S1 Relative taxa abundance comparisons at the phylum level between the COPD+ and COPD-,HIV+ and HIV-, andCOPD-HIV-, COPD-HIV+, COPD+HIV- and COPD+HIV+ groups in AEC samples. Table S2: Relative taxa abundance comparisons at the genus level between the COPD+ and COPD-, HIV+ and HIV-, and COPD-HIV-, COPD-HIV+, COPD+HIV- and COPD+HIV+ groups in AEC samples. Table S3: Pairwise PERMANOVA comparisons between the different specimen types obtained from HIV+ subjects. [file 12931_2023_2431_MOESM1_ESM.pdf]

Microbial Dysbiosis and the Host Airway Epithelial Response: Insights into HIV-Associated COPD Using Multi ’Omics Profiling

Additional file 1

Marcia Smiti Jude[1], Chen Xi Yang[1], Fernando Studart[1], Ana I Hernandez Cordero,[1] Julia Yang[1], Tawimas Shaipanich[2], Annie Li[1], David Lin[3], Julie MacIsaac[3], Michael S Kobor[3], Sunita Sinha[4], Corey Nislow[4], Amrit Singh[1], Silvia Guillemi[5,6], Marianne Harris[5,6], Julio Montaner[5,6], Raymond T Ng,[7] Christopher Carlsten[1,2], S. F. Paul Man[1,2], Don D Sin[1,2], Janice M Leung[1,2]

[1]Centre for Heart Lung Innovation, St. Paul’s Hospital, University of British Columbia, Vancouver, BC
[2]Division of Respiratory Medicine, Department of Medicine, University of British Columbia, Vancouver, BC
[3]Centre for Molecular Medicine and Therapeutics, University of British Columbia, Vancouver, BC
[4]Faculty of Pharmaceutical Sciences, University of British Columbia, Vancouver, BC
[5]British Columbia Centre for Excellence in HIV/AIDS, Providence Health Care, Vancouver, BC
[6]Faculty of Medicine, University of British Columbia, Vancouver, BC
[7]Department of Computer Science, University of British Columbia, Vancouver, BC, V6T1Z4, Canada.

Figure S1: Multi ‘omic integration study design. Figure created using BioRender.com.

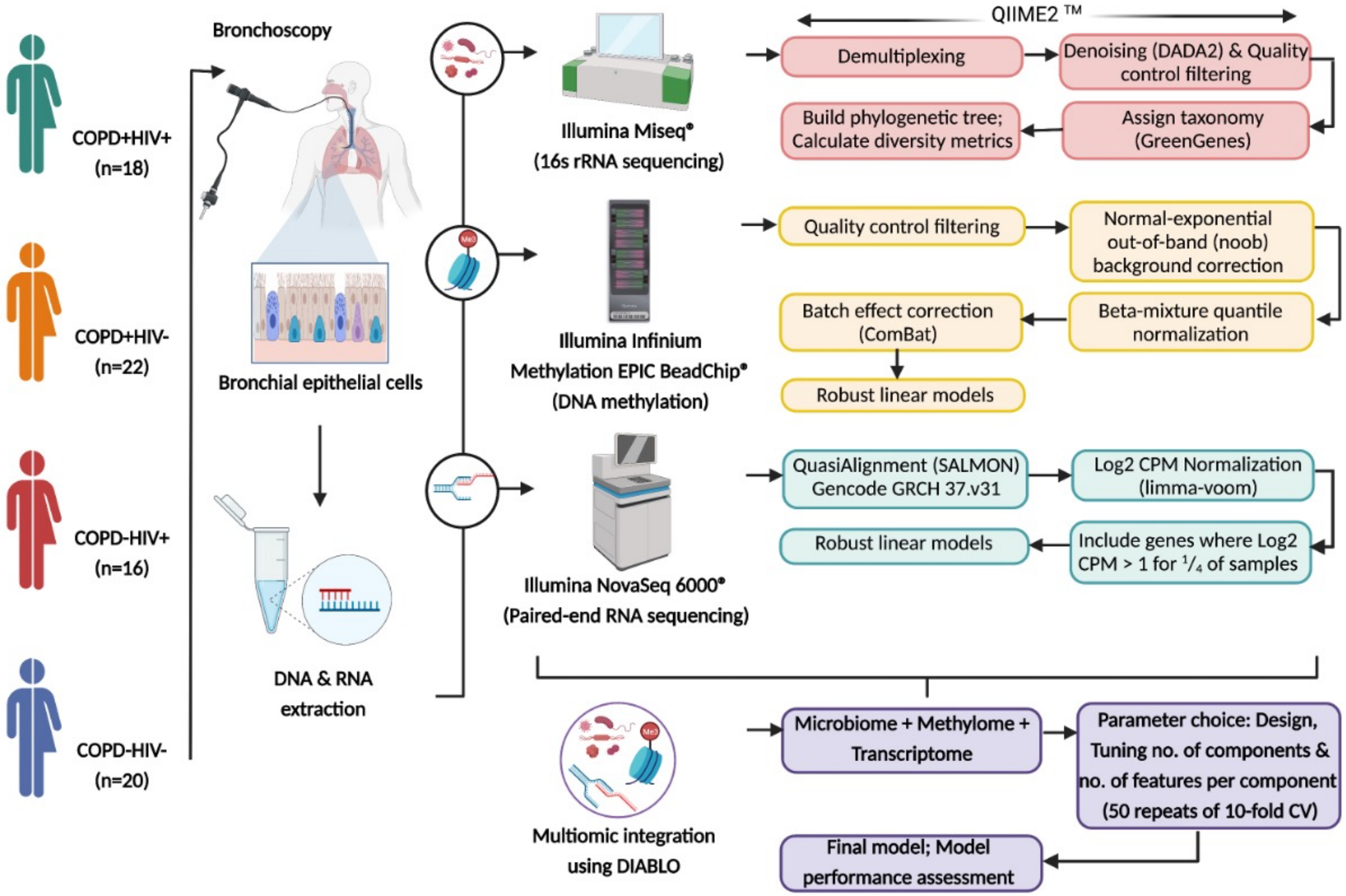

Figure S2: 16S RNA gene copies/µL measured in airway epithelial cells.

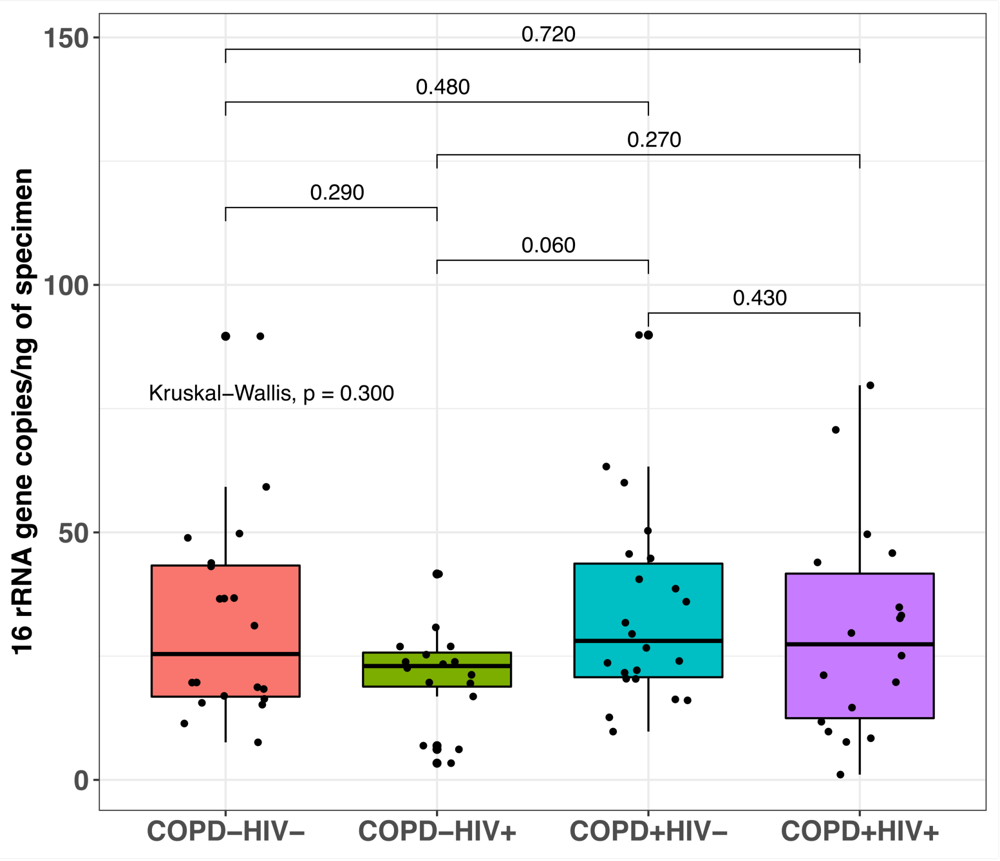

Figure S3: Principal coordinate plot by smoking status. Smoking status was significantly associated with beta diversity differences (p=0.037).

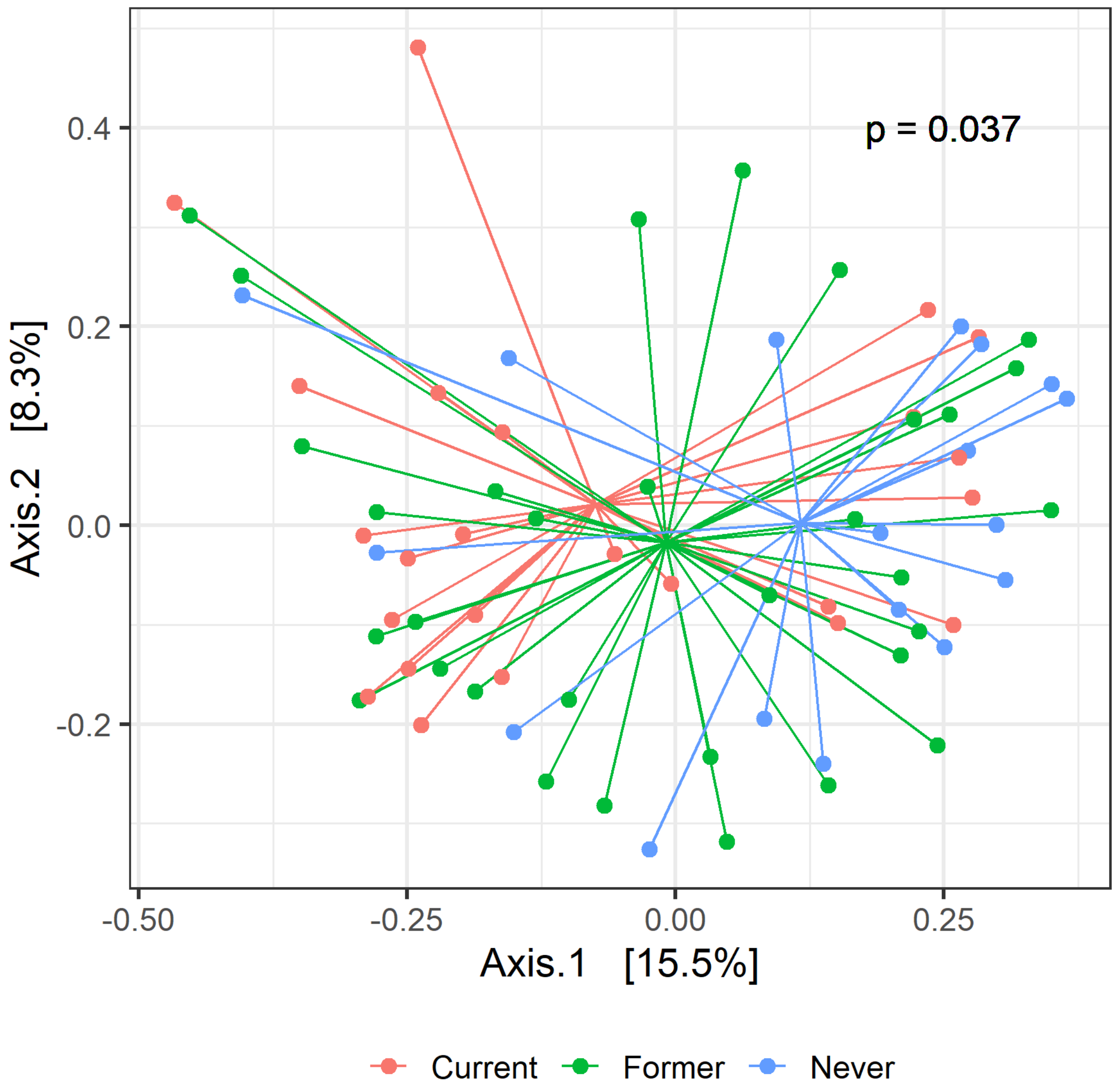

Figure S4: Plot showing potential interactive effects of COPD and HIV status on (a) Shannon Index, (b) Faith PD, (c) Bray Curtis PC1 (15.2% variance explained), and (d) Bray Curtis PC2 (8.5% variance explained). The lines are parallel in (a), (b) and (c) indicating the absence of an interaction effect. While the lines in (d) are intersecting indicating a potential interaction effect between COPD and HIV in PC2, no significant p-value was found.

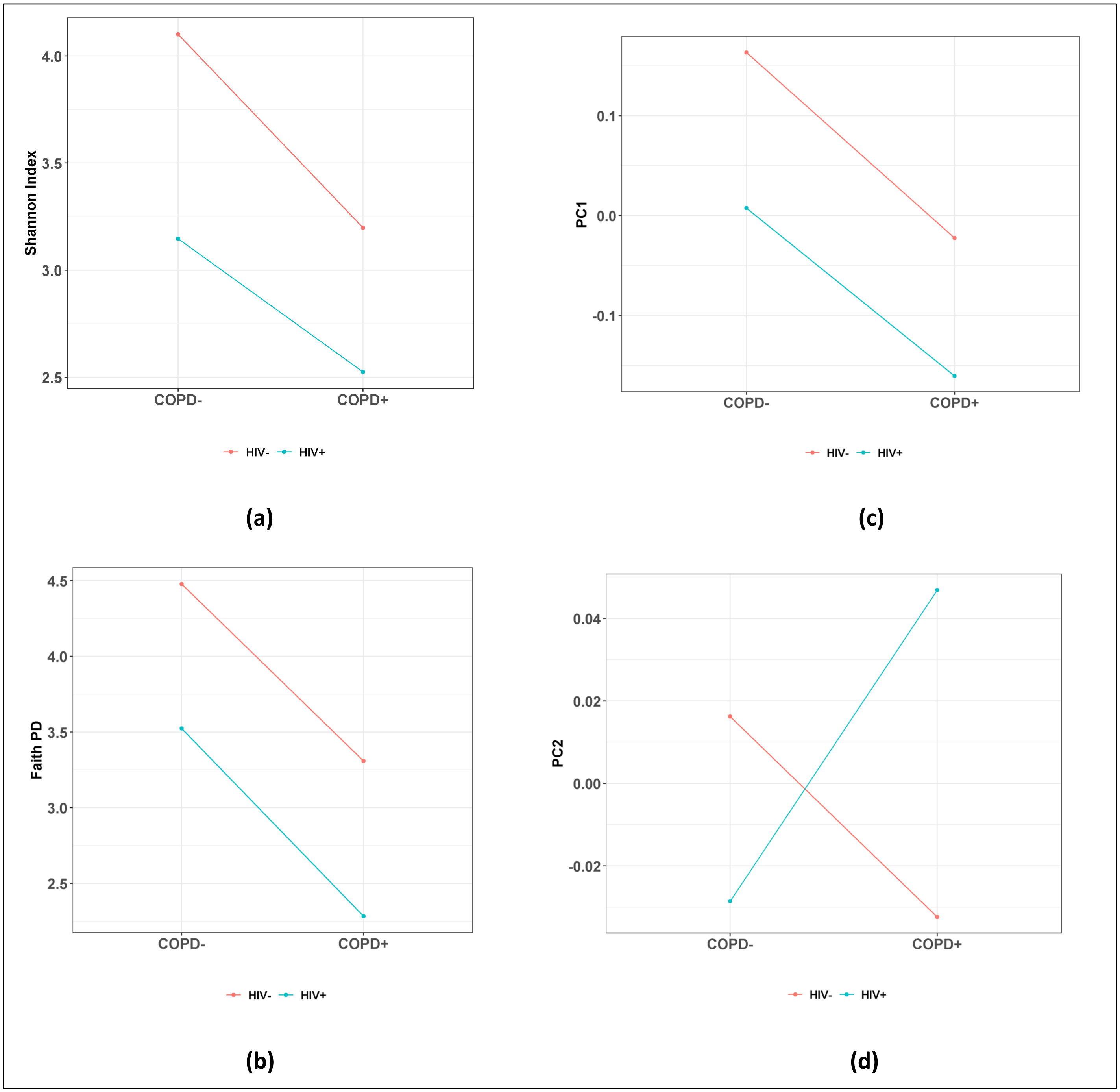

Figure S5: Shannon Diversity Index differences between the COPD and HIV groups (P= 2.9e-04) after removing 4 COPD- subjects administered with inhaled corticosteroids (ICS) from the analysis. No significant changes were observed in the results.

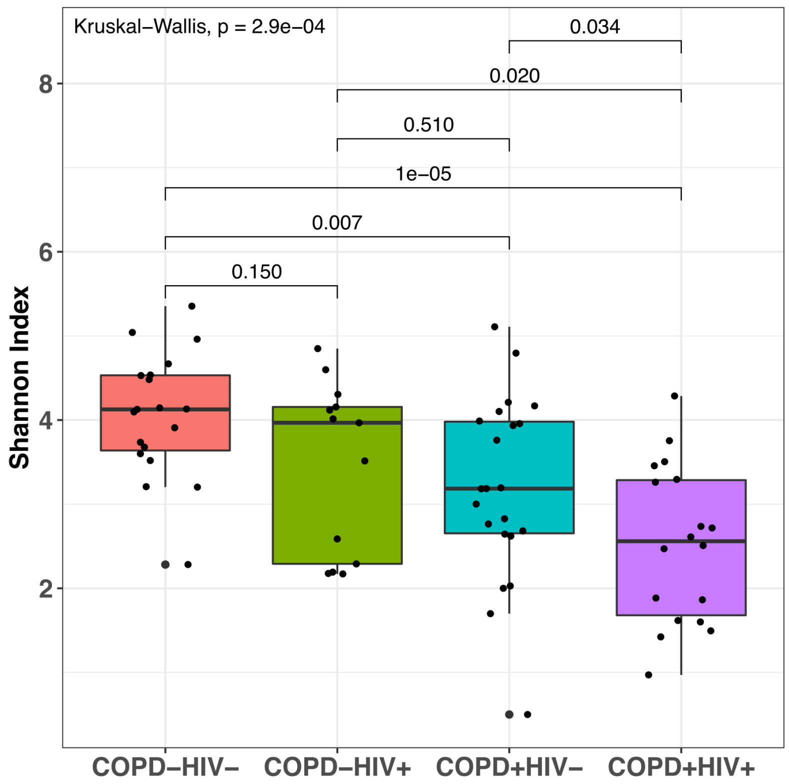

Figure S6: Principal coordinate plot showing microbial community structures among subject groups based on the Bray-Curtis metric, after removing 4 COPD- subjects administered with inhaled corticosteroids (ICS) from the analysis. Microbial community structures in AECs according to combined COPD+HIV status (COPD+HIV+ – purple points; COPD+HIV- – blue points; COPD-HIV+ – green points; COPD-HIV- – red points) based on Bray-Curtis distances; the centroids for each group are also shown. Definition of abbreviations: PC - principal component. No significant changes were observed in the results.

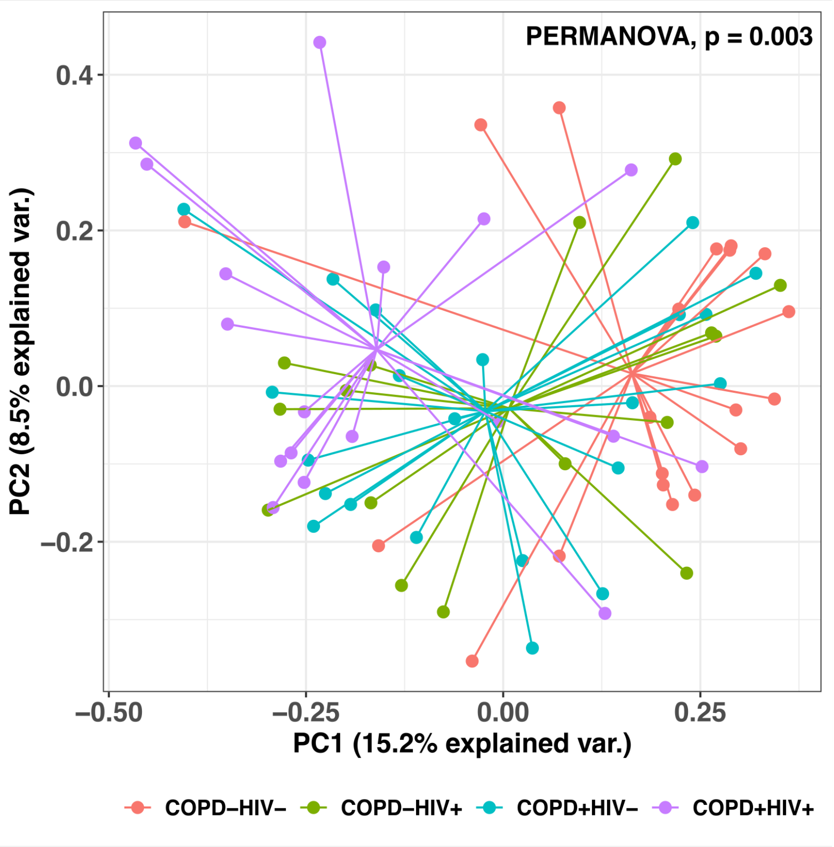

Figure S7: (a) 16S RNA gene copies/μL measured in bronchial brushings and control specimens of HIV+ subjects. Only significant Kruskal- Wallis pairwise comparison values are shown. (b) Principal coordinate plot showing microbial community structures among specimen types in HIV+ subjects based on the Bray-Curtis metric. The centroids for each group are also shown. Definitions of abbreviations: bronchoscope channel washes (BCW), brush water controls (BWC), cytolyt controls (CC), extraction negatives (EN), and oral wash controls (OWC)

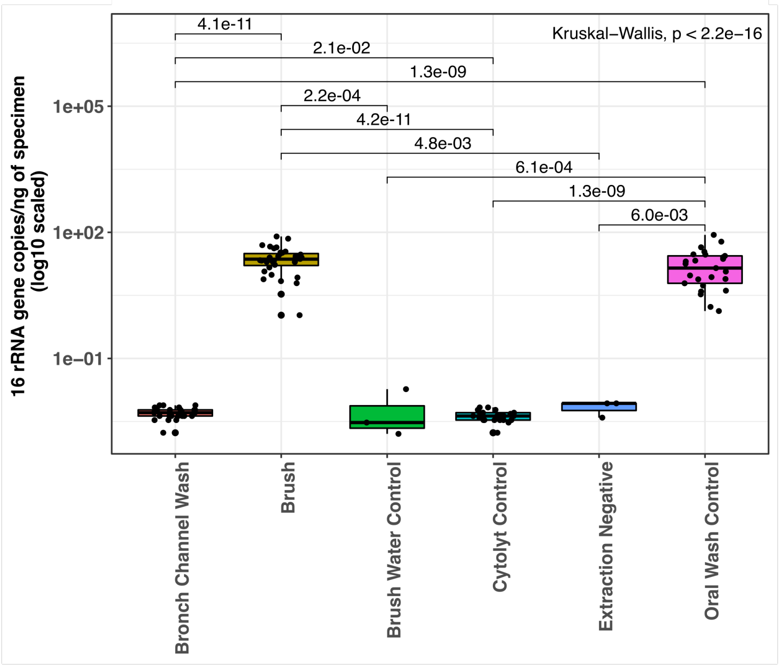

(a)

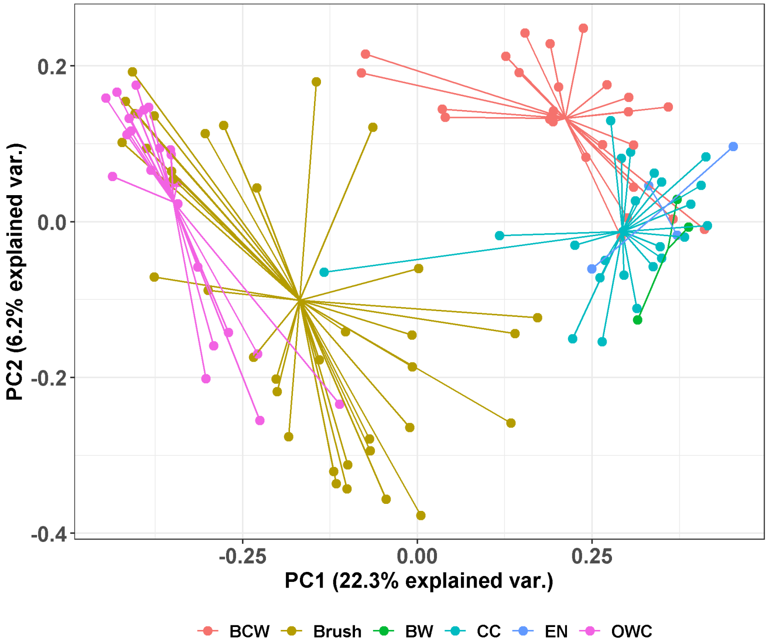

(b)

Figure S8: DIABLO circos plot showing the within and between correlations between the microbiome, transcriptome and methylome in the (a) COPD+ and COPD- groups, (b) HIV+ and HIV- groups, and (c) COPD*HIV groups. The three -omes are represented on the side quadrants; the level of each variable (ASV, CpG and gene) can be viewed along the circumference. Positive correlation - Green; Negative correlation - Red; Correlation cutoff = 0.7.

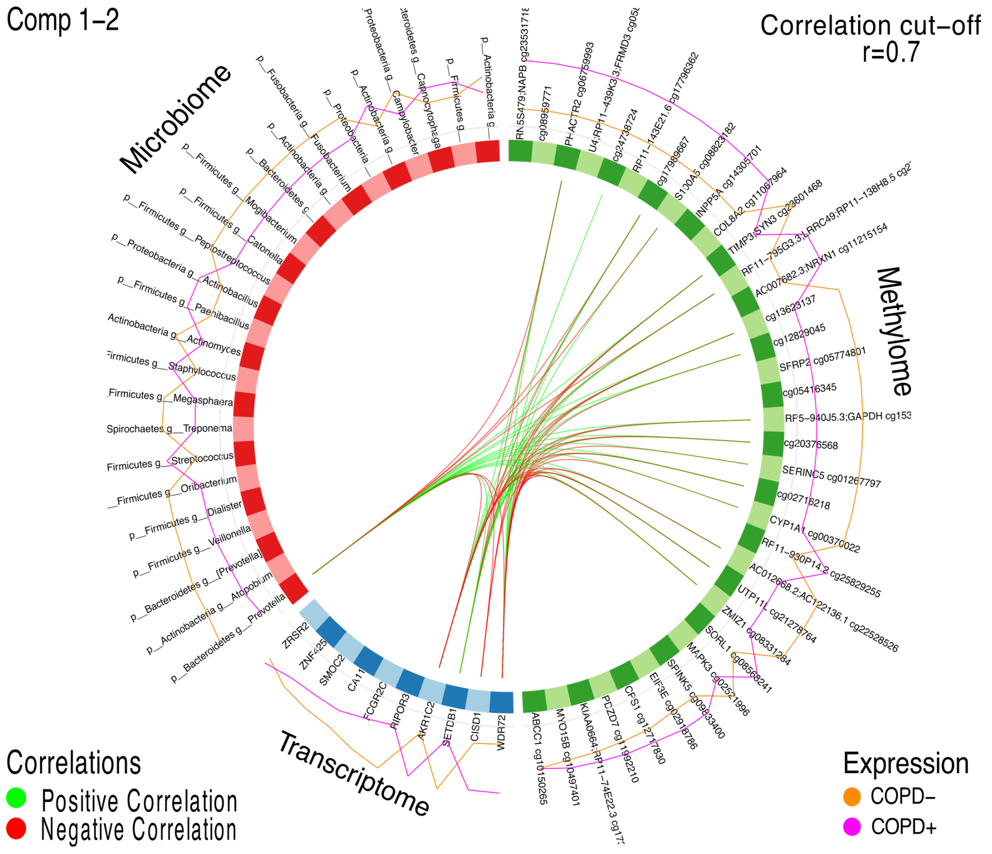

(a)

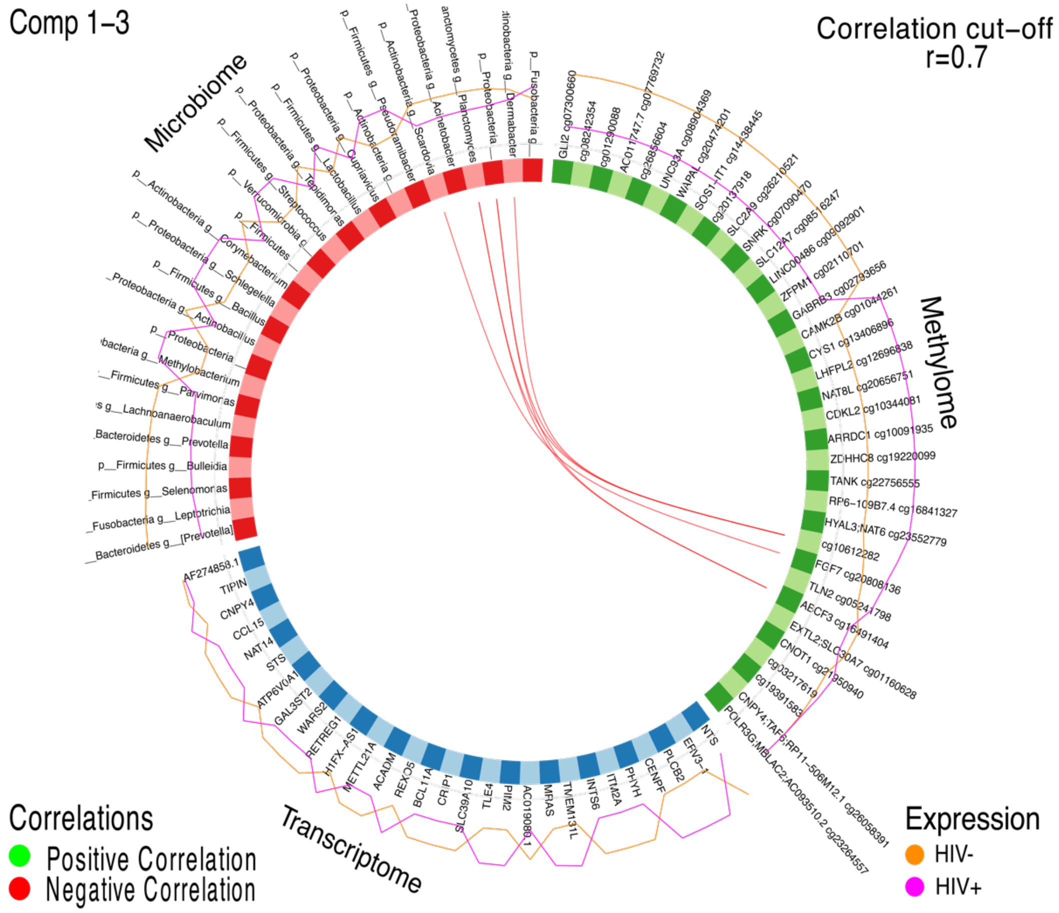

Comp 1–3
Correlation cut–off
r=0.7
Microbiome
Methylome
Transcriptome
Correlations
Positive Correlation
Negative Correlation
Expression
HIV-
HIV+

(b)

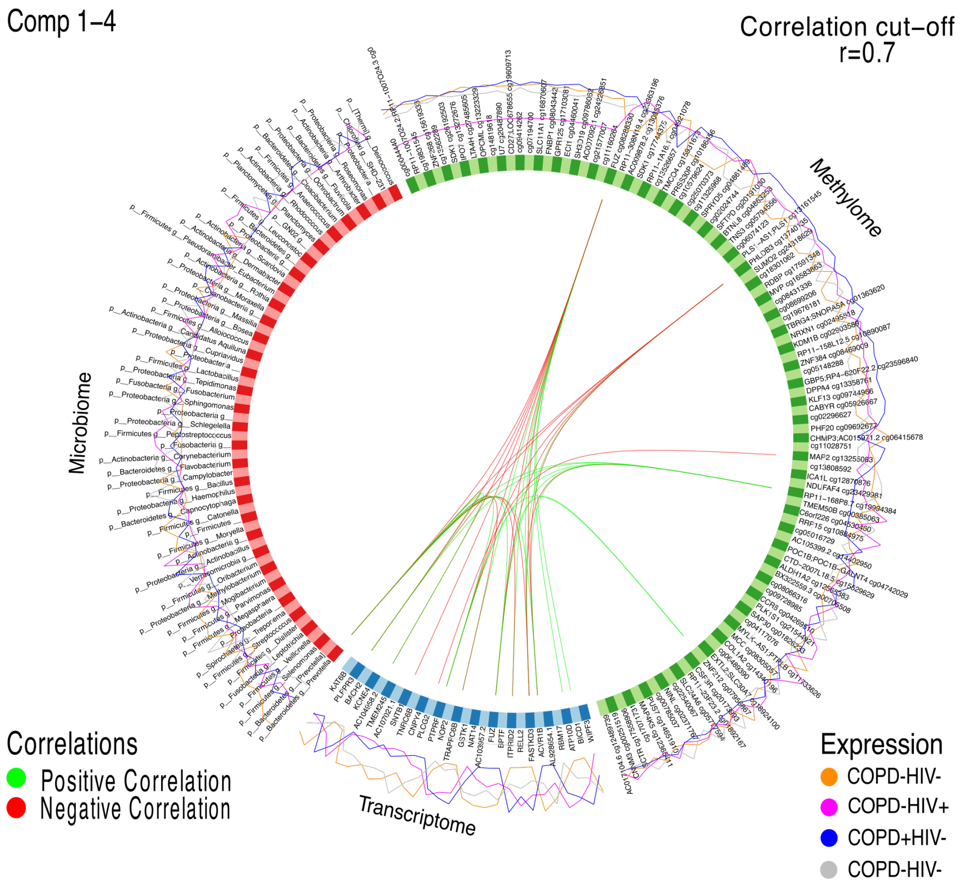
Comp 1–4
Correlation cut–off
r=0.7
Methylome
Microbiome
Transcriptome
Correlations
Positive Correlation
Negative Correlation
Expression
COPD-HIV-
COPD-HIV+
COPD+HIV-
COPD-HIV-

(c)

Table S1: (i) Relative taxa abundance comparisons at the phylum level between the (a) COPD+ and COPD-, (b) HIV+ and HIV-, and (c) COPD-HIV-, COPD-HIV+, COPD+HIV- and COPD+HIV+ groups in AEC samples. Values are displayed as median [interquartile range]. For (a) and (b), *P were calculated using the Mann–Whitney U-test; †Adjusted p were obtained using the Benjamini-Hochberg procedure (False Discovery Rate method). For (c) *P-values were calculated using the Kruskal-Wallis test; ±Effect size was calculated based on Kruskal-Wallis H-statistic [Eta squared method]. Definition of abbreviations: AEC – Airway epithelial cells.

Phylum	(a)COPD Effect				(b)HIV Effect			
	COPD- (n=36)	COPD+ (n=40)	*p	†Adjusted p	HIV- (n=42)	HIV+ (n=34)	*p	†Adjusted p
Firmicutes	0.386[0.221]	0.400[0.342]	0.357	0.536	0.362[0.19]	0.458[0.347]	0.123	0.247
Bacteroidetes	0.418[0.314]	0.185[0.355]	0.003	0.018	0.364[0.361]	0.203[0.365]	0.034	0.103
Proteobacteria	0.065[0.098]	0.169[0.246]	0.042	0.084	0.110[0.265]	0.080[0.179]	0.605	0.623
Actinobacteria	0.031[0.054]	0.037[0.067]	0.815	0.874	0.032[0.052]	0.038[0.071]	0.623	0.623
Fusobacteria	0.018[0.030]	0.000[0.025]	0.012	0.036	0.022[0.043]	0.000[0.011]	0.001	0.008
Verrucomicrobia	0.000[0.002]	0.000[0.006]	0.874	0.874	0.00[0.002]	0.000[0.016]	0.276	0.414

Phylum	(c) COPD*HIV Effect					
	COPD-HIV- (n=36)	COPD-HIV+ (n=40)	COPD+HIV- (n=36)	COPD+HIV+ (n=40)	*p	±Effect Size
Firmicutes	0.375[0.170]	0.386[0.379]	0.362[0.267]	0.541[0.359]	0.090	0.048[small]
Bacteroidetes	0.472[0.183]	0.273[0.352]	0.279[0.349]	0.107[0.333]	0.004	0.143[large]
Proteobacteria	0.067[0.116]	0.061[0.080]	0.232[0.287]	0.155[0.166]	0.198	0.023[small]
Actinobacteria	0.026[0.026]	0.054[0.081]	0.047[0.097]	0.022[0.048]	0.121	0.039[small]
Fusobacteria	0.030[0.044]	0.007[0.015]	0.009[0.044]	0.000[0.003]	0.0007	0.194[large]
Verrucomicrobia	0.000[0.002]	0.000[0.004]	0.000[0.002]	0.000[0.032]	0.523	-0.011[small]

(ii) Pairwise comparisons at the phylum level between the 4 COPD*HIV groups using Dunn's test (only significant comparisons displayed). †Adjusted p-values obtained on applying Bonferroni correction.

Group 1	Group 2	Phylum	*p	†Adjusted p
COPD-HIV-	COPD+HIV+	*Fusobacteria*<br>*Bacteroidetes*	0.0005<br>0.00033	0.0003<br>0.0019

Table S2: (i) Relative taxa abundance comparisons at the genus level between the (a) COPD+ and COPD-, (b) HIV+ and HIV-, and (c) COPD-HIV-, COPD-HIV+, COPD+HIV- and COPD+HIV+ groups in AEC samples.
Values are displayed as median [interquartile range]. For (a) and (b), *P were calculated using the Mann–Whitney U-test; †Adjusted p were obtained using the Benjamini-Hochberg procedure (False Discovery Rate method). For (c) *P-values were calculated using the Kruskal-Wallis test; ±Effect size was calculated based on Kruskal-Wallis H-statistic [Eta squared method]. Definition of abbreviations: AEC – Airway epithelial cells; *Prevotella∗ - Prevotella [f-Prevotellaceae], Prevotella∓ - Prevotella [f-Paraprevotellaceae]*.

Genus	(a)COPD Effect				(b)HIV Effect			
	COPD- (n=36)	COPD+ (n=40)	*p	†Adjusted p	HIV- (n=42)	HIV+ (n=34)	*p	†Adjusted p
Prevotella∗	0.334[0.302]	0.144[0.271]	0.0008	0.010	0.295[0.305]	0.127[0.315]	0.020	0.048
Veillonella	0.154[0.178]	0.065[0.156]	0.011	0.027	0.132[0.114]	0.112[0.268]	0.996	0.996
Streptococcus	0.057[0.060]	0.094[0.196]	0.233	0.028	0.067[0.081]	0.067[0.164]	0.627	0.752
Haemophilus	0.015[0.049]	0.001[0.068]	0.157	0.209	0.013[0.054]	0.002[0.051]	0.354	0.472
Paenibacillus	0.000[0.003]	0.005[0.020]	0.028	0.042	0.000[0.012]	0.001[0.020]	0.817	0.892
Rothia	0.008[0.025]	0.006[0.026]	0.680	0.742	0.010[0.021]	0.002[0.028]	0.105	0.158
Actinobacillus	0.000[0.000]	0.000[0.000]	0.980	0.980	0.000[0.000]	0.000[0.000]	0.030	0.061

Megasphaera	0.012[0.046]	0.000[0.008]	0.005	0.024	0.008[0.036]	0.000[0.007]	0.071	0.122
Prevotella∓	0.012[0.043]	0.000[0.011]	0.008	0.024	0.012[0.042]	0.000[0.001]	0.0003	0.004
Neisseria	0.002[0.019]	0.000[0.004]	0.158	0.032	0.002[0.016]	0.000[0.002]	0.017	0.048
Selenomonas	0.002[0.018]	0.000[0.001]	0.020	0.035	0.001[0.039]	0.000[0.001]	0.014	0.048
Fusobacterium	0.008[0.027]	0.000[0.015]	0.007	0.024	0.010[0.027]	0.000[0.006]	0.012	0.048

Genus	(c) COPD*HIV Effect					
	COPD-HIV- (n=36)	COPD-HIV+ (n=40)	COPD+HIV- (n=36)	COPD+HIV+ (n=40)	*p	±Effect Size
Prevotella*	0.414[0.151]	0.228[0.308]	0.214[0.286]	0.024[0.240]	0.0008	0.191[large]
Veillonella	0.157[0.106]	0.149[0.221]	0.073[0.119]	0.040[0.256]	0.077	0.0535[small]
Streptococcus	0.054[0.053]	0.061[0.147]	0.080[0.140]	0.140[0.253]	0.588	-0.0150[small]
Haemophilus	0.015[0.046]	0.014[0.048]	0.005[0.069]	0.000[0.055]	0.331	0.0059[small]

Paenibacillus	0.000[0.005]	0.000[0.001]	0.001[0.014]	0.009[0.064]	0.024	0.0901[moderate]
Rothia	0.008[0.011]	0.004[0.040]	0.012[0.050]	0.001[0.023]	0.401	-0.0008[small]
Actinobacillus	0.000[0.000]	0.000[0.000]	0.000[0.000]	0.000[0.000]	0.100	0.0452[small]
Megasphaera	0.032[0.054]	0.005[0.037]	0.000[0.022]	0.000[0.000]	0.011	0.112[moderate]
Prevotella ∓	0.026[0.041]	0.000[0.016]	0.004[0.021]	0.000[0.000]	0.0002	0.236[large]
Neisseria	0.003[0.027]	0.000[0.009]	0.000[0.007]	0.000[0.000]	0.009	0.119[moderate]
Selenomonas	0.005[0.046]	0.000[0.004]	0.000[0.024]	0.000[0.000]	0.010	0.116[moderate]
Fusobacterium	0.025[0.032]	0.003[0.010]	0.002[0.020]	0.000[0.001]	0.003	0.155[large]

(ii) Pairwise comparisons at the genus level between the 4 COPD*HIV groups using Dunn's test (only significant comparisons displayed). †Adjusted p-values obtained on applying Bonferroni correction. ***Prevotella∗ - Prevotella [f- Prevotellaceae], Prevotella∓ - Prevotella [f-Paraprevotellaceae].***

Group 1	Group 2	Phylum	*p	†Adjusted p
COPD-HIV-	COPD+HIV+	*Prevotella∓*<br>*Prevotella∗*<br>*Fusobacterium*<br>*Selenomonas*<br>*Neisseria*<br>*Megasphaera*	0.00001<br>0.00007<br>0.0003<br>0.0007<br>0.0007<br>0.001	0.00007<br>0.0004<br>0.002<br>0.004<br>0.004<br>0.007
COPD-HIV-	COPD+HIV-	*Prevotella∗*	0.0052	0.031
COPD-HIV-	COPD-HIV+	*[Prevotella]*	0.006	0.034
COPD-HIV+	COPD+HIV+	*Paenibacillus*	0.002	0.013

Table S3: Pairwise PERMANOVA comparisons between the different specimen types obtained from HIV+ subjects. †Adjusted p obtained using the Benjamini-Hochberg procedure (False Discovery Rate method). Definition of abbreviations: BCW - bronchoscope channel wash; BW - brush water control; CC - cytolyt control; EN - extraction negative; OWC - oral wash control.

Group 1	Group 2	Sample size	p-value	† Adjusted p
BCW	BW	28	0.002	0.003
	Brush	101	0.001	0.002
	CC	49	0.001	0.002
	EN	29	0.044	0.055
	OWC	50	0.001	0.002
BW	Brush	79	0.001	0.002
	CC	27	0.634	0.732
	EN	7	0.767	0.822
	OWC	28	0.001	0.002
Brush	CC	100	0.001	0.002
	EN	80	0.001	0.002
	OWC	101	0.001	0.002
CC	EN	28	0.954	0.954
	OWC	49	0.001	0.001
EN	OWC	29	0.001	0.002

Supplementary Methods

Methylome Profiling:

DNA methylation in bronchial brushings was profiled using the Illumina Infinium Methylation EPIC BeadChip®. Downstream quality filtering, normalization, and background and batch-effect correction steps were performed. PCA was used to compare distances between the respective groups in the independent COPD, HIV, and COPD*HIV effect analyses **(Supplementary Methods Figure 1)**. Covariate selection analysis was performed to adjust for any confounding variables that may have a significant effect on methylation - none were identified **(Supplementary Methods Figure 2).** To identify significantly differentially methylated probes (DMPs) or CpG sites, the following robust linear models were used:

Methylation (M value) ~ COPD status + EPISTRUCTURE PC1–PC5

Methylation (M value) ~ HIV status + EPISTRUCTURE PC1–PC5

*Methylation (M value) ~ COPD*HIV status + EPISTRUCTURE PC1–PC5*

Top differentially methylated positions (DMPs) and regions (DMRs) at FDR < 0.05 identified: 8,736 DMPs and 1,110 DMRs in the COPD analysis, 61,536 DMPs and 6,417 DMRs in the HIV analysis, and 1,755 DMPs and 211 DMRs in the COPD*HIV analysis.

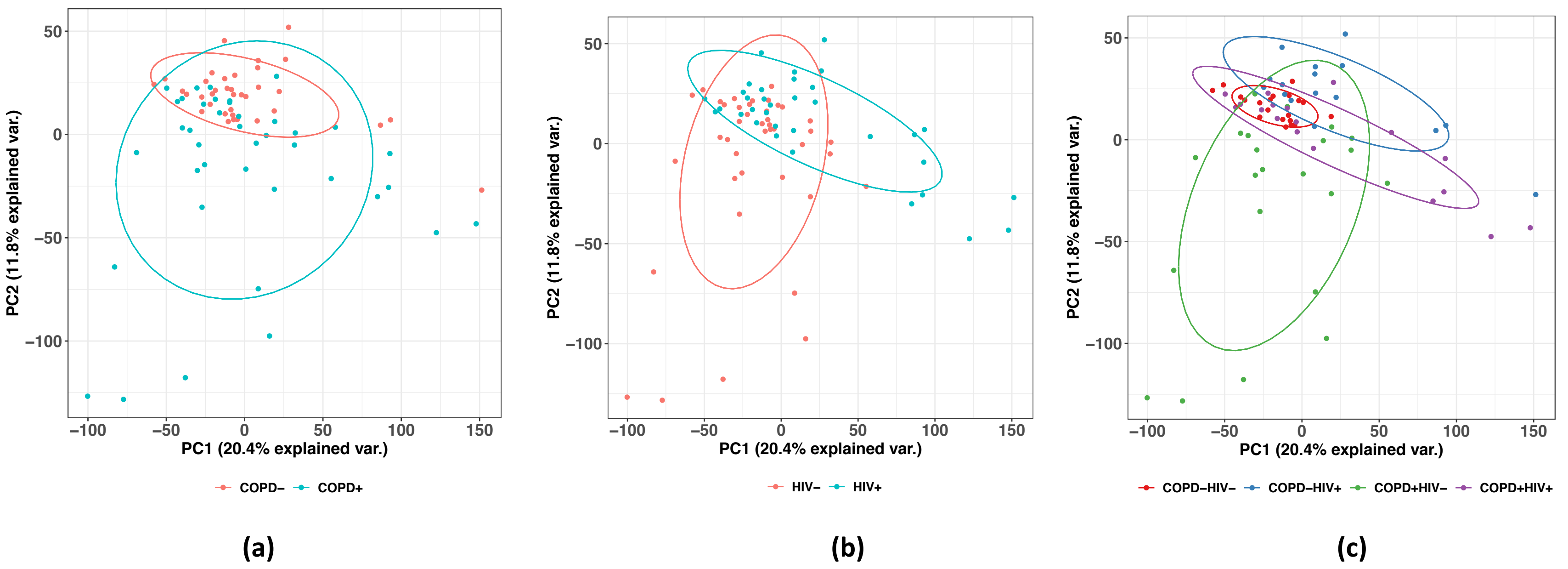

Supplementary Methods Figure 1: Principal component analysis of methylation profiles according to (a) COPD, (b) HIV and (c) combined COPD*HIV status. (a) COPD status (COPD- patients (N) – red points; COPD+ patients (Y) – blue points), (b) HIV status (HIV- patients (Negative) – red points; HIV+ patients (Positive) – blue points), and (c)combined COPD*HIV status (COPD+HIV+ – purple points; COPD+HIV- – blue points; COPD-HIV+ – green points; COPD-HIV- – red points); the ellipses enclosing each group are also shown. Definition of abbreviations: PC - principal component.

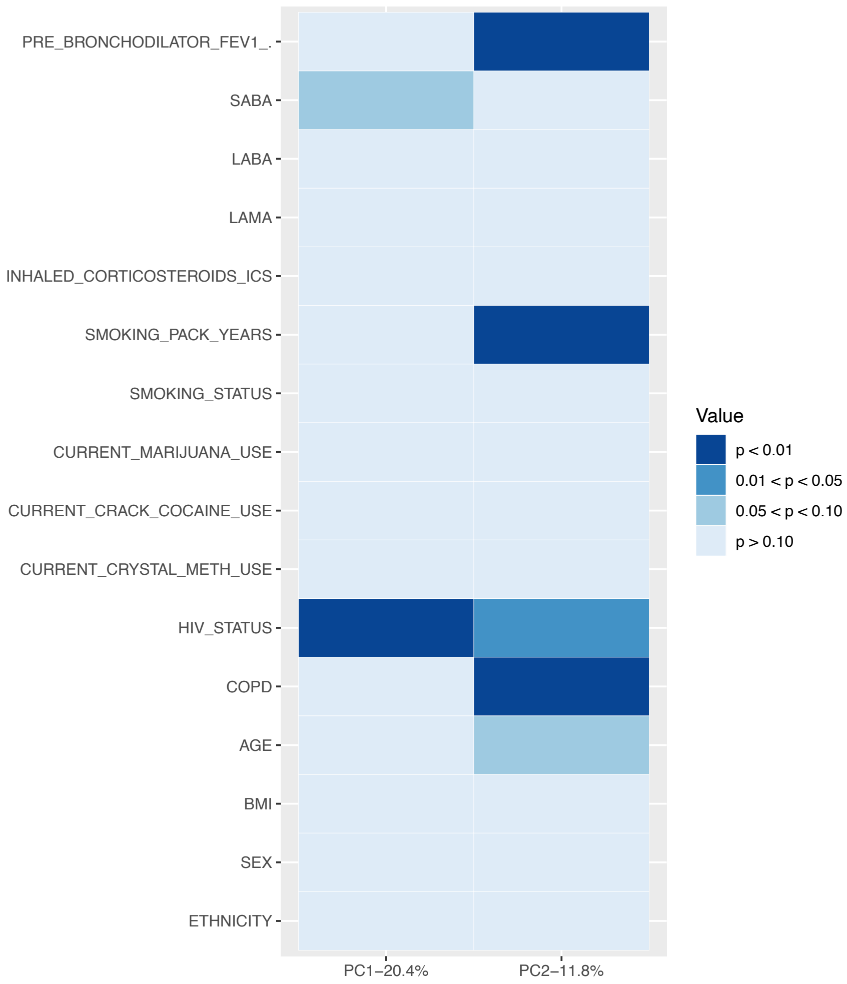

Supplementary Methods Figure 2: Covariate selection for methylome analysis.

Heatmap of principal components (PCs) vs. covariates of interest (ethnicity, sex, age, body-mass index (BMI), pre-bronchodilator FEV1%, drug use, smoking status, smoking pack years, inhaled corticosteroid (ICS), long-acting muscarinic antagonists (LAMA), long-acting beta antagonists (LABA) and short-acting beta antagonists (SABA) use, COPD and HIV status). The color scale represents p-values, grey indicating low level and navy indicating high level of significance.

Transcriptome Profiling:

RNA extracted from the bronchial brushings was sequenced using the Illumina NovaSeq 6000®RNA sequencing system. This was followed by quality filtering, paired-end read alignment, batch effect analysis, and normalization. PCA was used to visualize gene expression patterns among the COPD, HIV and COPD*HIV groups are included in **Supplementary Methods Figure 3.** Covariate selection analysis identified one variable that may have a significant effect on gene expression - Sex **(Supplementary Methods Figure 4).** To identify the top differentially expressed genes the following robust linear models (on adjusting for *SEX*) were used:

Gene expression $\sim$ *COPD status + Sex*

Gene expression $\sim$ *HIV status + Sex*

Gene expression $\sim$ *COPD*HIV status + Sex*

Robust linear regression (correcting for sex) identified 6,033, 274 and 28 differentially expressed genes (DEGs) with false discovery rate (FDR) < 0.1 in the COPD, HIV, and COPD*HIV analysis, respectively.

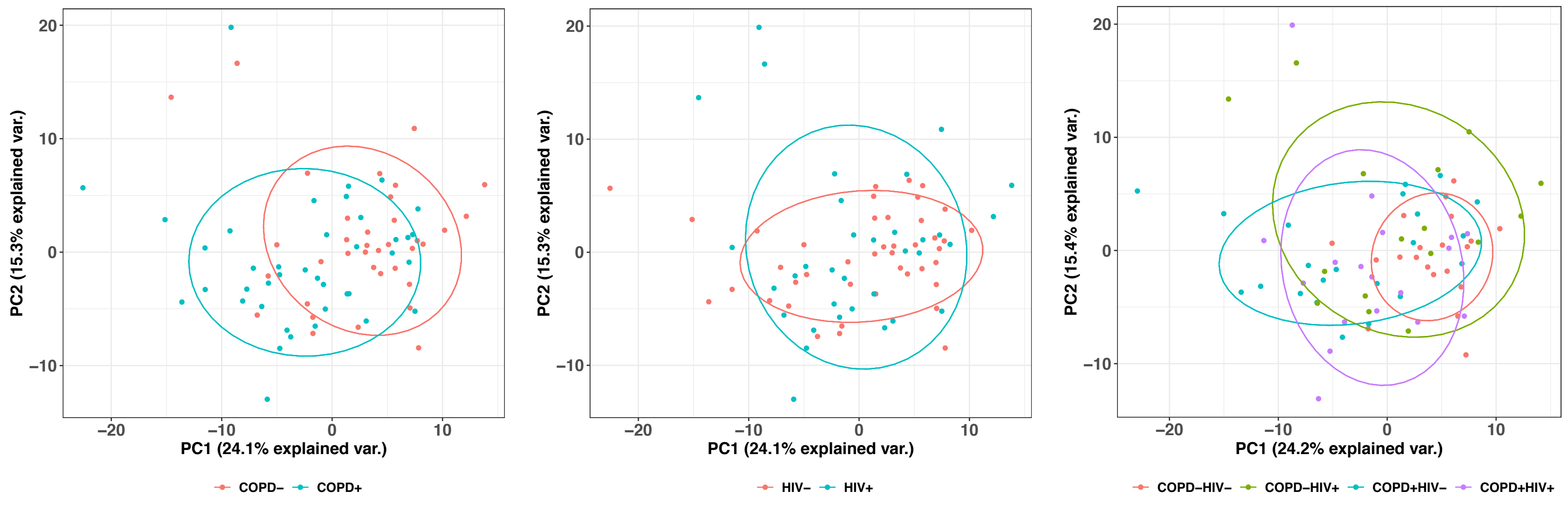

(a) (b) (c)

Supplementary Methods Figure 3: Principal components analysis of gene expression profiles according to (a) COPD, (b) HIV and (c) combined COPD*HIV status.

(a) COPD status (COPD- patients (N) – red points; COPD+ patients (Y) – blue points), (b) HIV status (HIV- patients (Negative) – red points; HIV+ patients (Positive) – blue points), and (c) combined COPD*HIV status (COPD+HIV+ – purple points; COPD+HIV- – blue points; COPD-HIV+ – green points; COPD-HIV- – red points); the ellipses enclosing each group are also shown)

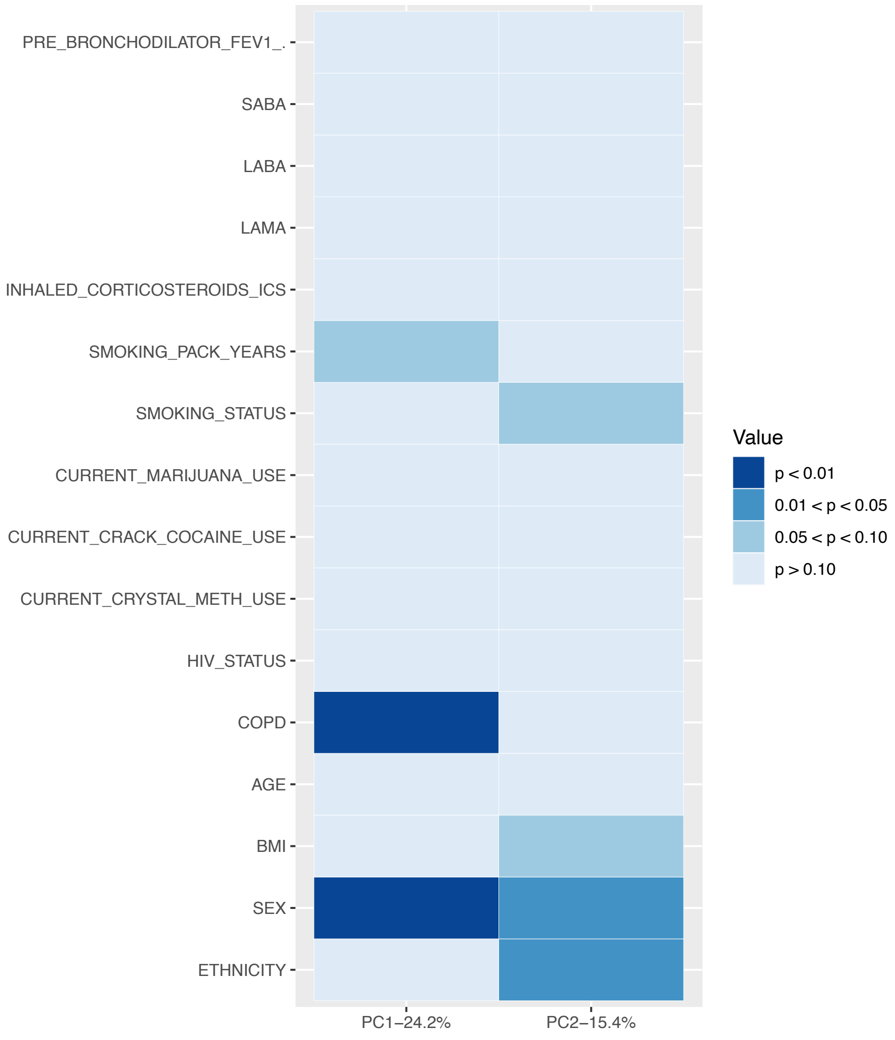

Supplementary Methods Figure 4: Covariate selection for transcriptome analysis.

Heatmap of principal components (PCs) vs. covariates of interest (ethnicity, sex, age, body-mass index (BMI), pre-bronchodilator FEV1% predicted, drug use, smoking status, smoking pack years, inhaled corticosteroid (ICS), long-acting muscarinic antagonists (LAMA), long-acting beta antagonists (LABA) and short-acting beta antagonists (SABA) use, COPD and HIV status). The color scale represents p-values, grey indicating low level and navy indicating high level of significance.
